# Dental Periostitis—The Grindstone Cure

**Published:** 1883-10

**Authors:** Jos. Requa

**Affiliations:** Rochester, N. Y.


### DENTAL PERIOSTITIS—THE GRINDSTONE CURE.

BY JOS. REQUA, ROCHESTER, N. Y.

READ BEFORE THE 7TH AND 8TH DISTRICT DENTAL SOCIETIES, OCTOBER, 1882.

A peculiar phase of periostitis, with accompanying discomfort and toothache, has so forced itself upon my attention the past year and has been so interesting to me, that I wish to call your attention to the subject, and will do so by giving the history of a few cases.

The teeth and their surroundings to be maintained in health need exercise, such as they get or ought to get in the mastication of food. In order to get this exercise they must have antagonists. Usually when a tooth has lost its antagonist we see it slowly and surely losing its firmness and becoming elongated, or leaving its socket.

On the other hand it must not be subjected to immoderate usage and pressure, such as a molar or bicuspid would get if we were to fill the crown with gold or other hard substance and leave it too full, or such as it would get if an individual tooth were harder than the other and thus less subject to wear by attrition.

Now there is nothing new in all this, and yet I fear there are some among us who do not give sufficient attention to—if I may be allowed the expression—the harmonious antagonism of the teeth, especially in cases of obscure pain.

A year ago a lady of middle age, with full compliment of teeth containing several fillings, all of which were gold, complained of uncomfortable feeling in the region of the upper left molars. I examined them but failed to find the cause. A week later she came in with "there must be a cavity in one of these teeth, as hot and cold liquids give me severe pain." I spent half an hour in a further examination. The verdict was—no cause for action. A week later and a draught of air through the mouth gave pain. Then for the first time I found sensitiveness to precussion in one of the bicuspids. I suspected a dead pulp, as it contained a good filling of fifteen years standing. A few days more showed marked periostitis. From a bite upon sheet wax I found the tooth receiving hard knocks from a hard, unworn cusp of its antagonist. I ground away the hard-headed antagonist with instant and permanent relief, and there has been no discomfort whatever since the hyper-sensitive tooth was relieved of more than its share of pressure.

Four years ago I removed the pulp from a lower molar for a man of about fifty years, and filled the roots with os-artificial and the crown with amalgam. Last January he came in saying: "That tooth will have to come out now." He had lost but two or three teeth, and the others showed extensive wear excepting this one. By a bite upon sheet wax I found it to be receiving more pressure than the others; it was a severe case of periostitis, and increasing daily. I ground the tooth so that it scarcely touched, and he remarked, "There, that is all right," and it has remained all right to the present time.

In February last a young man applied for relief. He had lain awake nights, and suffered daily after eating, for more than a week. I could not locate the pain, as it was neuralgic in character, but after several days had passed, during which I made four or five examinations, a lower second molar began to grow sensitive to percussion. It had been filled seven or eight years with amalgam, and now showed a cleaner cut through sheet wax than its neighbors. I applied the grindstone cure, and there has been no discomfort since.

In February a man of forty-five who had been unable to attend to business for a week because of a sore tooth, came to have it out. A lower second bicuspid was jumping up and down in its socket, and I at first thought there was alveolar abscess. A further examination led me to think there was not, and that the pulp was alive. I applied the grindstone cure, with immediate relief and a rapid return to health. He was employed in the city treasurer's office, and it was a plain case of unequal taxation, and the overtaxed member grumbled, as it had an inalienable right to do.

Between two and three weeks ago, a man of fifty who lives in Chicago, but who was spending a few days in our city, called and wished me to examine an upper second molar. It had troubled him a good deal for three or four years. He had thirty-one teeth, all nearly sound, three or four small fillings in them, and an amalgam filling on the posterior side of the troublesome one. The wisdom tooth back of it had been extracted about four years before. He said it was not sensitive to heat or cold, but most painful after eating. His dentist said he could do nothing except to extract it. I found the filling perfect, the tooth otherwise sound, and the gum apparently healthy. I ground it and its antagonist, and requested him to report the result. I have not seen him since, but a week afterward received a postal card saying his tooth was all right, and that he had had no pain since the application of our specific.

Three months ago a lady began troubling me with complaints of toothache, for which I could find no cause. The trouble gradually increased until about a month ago, when she came in, saying she had suffered so much that she could bear it no longer, and that cold water gave excruciating pain. With a syringe and cold water I soon found the offender to be an upper second molar, in which an approximal filling of phosphate of zinc had been inserted a year or two before. The filling was in good condition. I ground the tooth so that it did not cut through sheet wax. I did not see her again until yesterday, when she came in smiling all over, saying she thought it wonderful how such a simple thing had cured her of the "awfulest toothache" she had ever had.

These are only a few of the many cases, but sufficient to show the point I wish to make. We do know that teeth often differ individually in the same mouth in structure and density, with corresponding differences in their ability to withstand wear. As a general rule, however, they accommodate themselves to circumstances, and keep up the balance of antagonism unassisted; yet I have been astonished at the number of cases I have found since I have been looking for them, where a little assistance was needed, and which when rendered, met the requirements of the case perfectly.

